# A modified dynamic DEA model to assess the wastewater treatment efficiency: perspective from Yangtze River and Non-Yangtze River Basin

**DOI:** 10.1038/s41598-022-14105-0

**Published:** 2022-06-15

**Authors:** Fangrong Ren, Yanan Sun, Jiawei Liu, Kejing Chen, Naixin Shi

**Affiliations:** 1 College of Economics and Management, Nanjing Forestry University, Nanjing, 210037 People’s Republic of China; 2 School of Economics and Management, Nantong University, No. 9 Seyuan Road, Nantong, 226019 Jiangsu People’s Republic of China; 3 Business School, Hohai University, No. 8 Focheng West Road, Nanjing, 211100 People’s Republic of China

**Keywords:** Environmental sciences, Environmental social sciences

## Abstract

The wastewater treatment efficiency is crucial to constructing a livable ecological environment and promoting the sustainable development of economy and society. The differences in natural conditions, economic development and local policies between the Yangtze River Basin (YRB) and the Non-Yangtze River Basin (NYRB) increase the difficulty of wastewater treatment in governance. This study uses a modified Dynamic Data Envelopment Analysis (DEA) model to assess the wastewater treatment from 2013 to 2020, and divides the study period into two stages: the first stage (2013–2017) assesses the wastewater treatment efficiency of 18 provinces and cities in YRB and 12 provinces and cities in NYRB; the second stage (2018–2020) conducts statistical analysis of wastewater discharge pollutants in YRB and NYRB. The results conclude that the total wastewater treatment efficiency is generally low, but polarization is quite prominent. Among total wastewater treatment efficiency, NYRB scored 0.504, or slightly higher than YRB (0.398). In terms of expense efficiency, both NYRB and YRB scored below 0.4. In terms of chemical oxygen demand (COD) output efficiency, YRB (0.488) is better than NYRB (0.420). The second stage of statistical analysis presents that pollutant emissions are still high; the regions need to increase wastewater treatment investment and improve wastewater treatment efficiency.

## Introduction

Water pollution has long been a serious environmental challenge for countries all over the world^1–3^. As China’s process of industrialization and urbanization keeps accelerating, water demand continues to grow, and the contradiction between water supply and demand is turning increasingly prominent. China has a large population, but less water resources per capita. Its temporal and spatial distributions of water resources are also uneven. In addition, the whole society does not have a strong water-saving consciousness, given the extensive use of water and serious waste of it that result in the inefficient use of water resources and the discharge of a large amount of wastewater.

According to statistics released by the Ministry of Housing and Urban–Rural Development, the annual discharge of urban sewage in China has increased year by year in the past decade. In 2010, it was only 37.87 billion cubic meters, but in 2016 the annual discharge of sewage hit 48.030 billion cubic meters, exceeded 50 billion cubic meters in 2018, increased to 57.136 billion cubic meters in 2020, and was forecasted to reach 58.964 billion cubic meters in 2021^4^. A large amount of wastewater pollution has caused serious water pollution in rivers and lakes. At the same time, sixteen of China’s 31 provinces are facing water shortages, 300 cities are short of water to varying degrees, and the direct loss caused by water shortages is 200 billion yuan every year^5^. Thus, the China government has attached great importance to the development of the wastewater treatment industry, promulgated a series of policies and regulations^6^, such as “10-point Water Plan”^7^, “River Chief policy”^8^, “Water Emissions Trading policy”^9^, put forward the goal of “comprehensive improvement of ecological environment quality”, and raised the concept of ecological civilization to the level of its national reform and development strategy.

The Yangtze River Basin (YRB) is the third largest river basin in the world with a drainage area of 1.8 million square kilometers and refers to the vast area where the main stream and tributaries of the Yangtze River flow. Average annual water resources amount to 995.8 billion m^3^, accounting for about 35% of total national water resources. However, with the rapid development of the economy and society in recent years, the ecological environment protection situation in YRB is not optimistic. In the case of the Yangtze River Economic Zone, although it only accounts for 21% of the country, according to years of data monitoring, its total wastewater discharge is over 40% of the country’s total, and chemical oxygen demand, ammonia nitrogen, sulfur dioxide, nitrogen oxides, and volatile organic compound emissions per unit area are 1.5–2 times the intensity of the national average. There is more to consider about the problem of treating wastewater in YRB, because it involves many administrative divisions and broader local protectionism. But some inland provinces in Non-Yangtze River Basin (NYRB) have weak social and economic foundation, lack of policy support and financial strength for wastewater treatment in YRB, and sewage treatment legislation is imminent^6^. At the same time, the differences in natural conditions, economic development and local policies between the YRB and NYRB also lead to difficulties in governance. Therefore, it is necessary to study the difference of wastewater treatment efficiency between YRB and NYRB.

In order to quantify the dynamic evolution of wastewater treatment efficiency in YRB and NYRB, it is necessary to observe wastewater treatment efficiency at the provincial level on a long-time scale. DEA method has already been widely used in the efficiency measurement of wastewater treatment^10–15^, wastewater water treatment plants^16–18^, energy^19–21^, environment^22–25^, and other aspects. Compared with other DEA methods, Dynamic DEA method can more effectively reflect the real efficiency of Decision Making Unit (DMU) by describing and analyzing the internal structure of the system and considering the continuity of multi-cycle operation of the system. On this basis, this research constructs a modified Dynamic DEA model by considering undesired outputs and employs statistical analysis to evaluate the wastewater treatment efficiency and treatment status in 30 provinces from 2013 to 2020.

**Figure 1:**
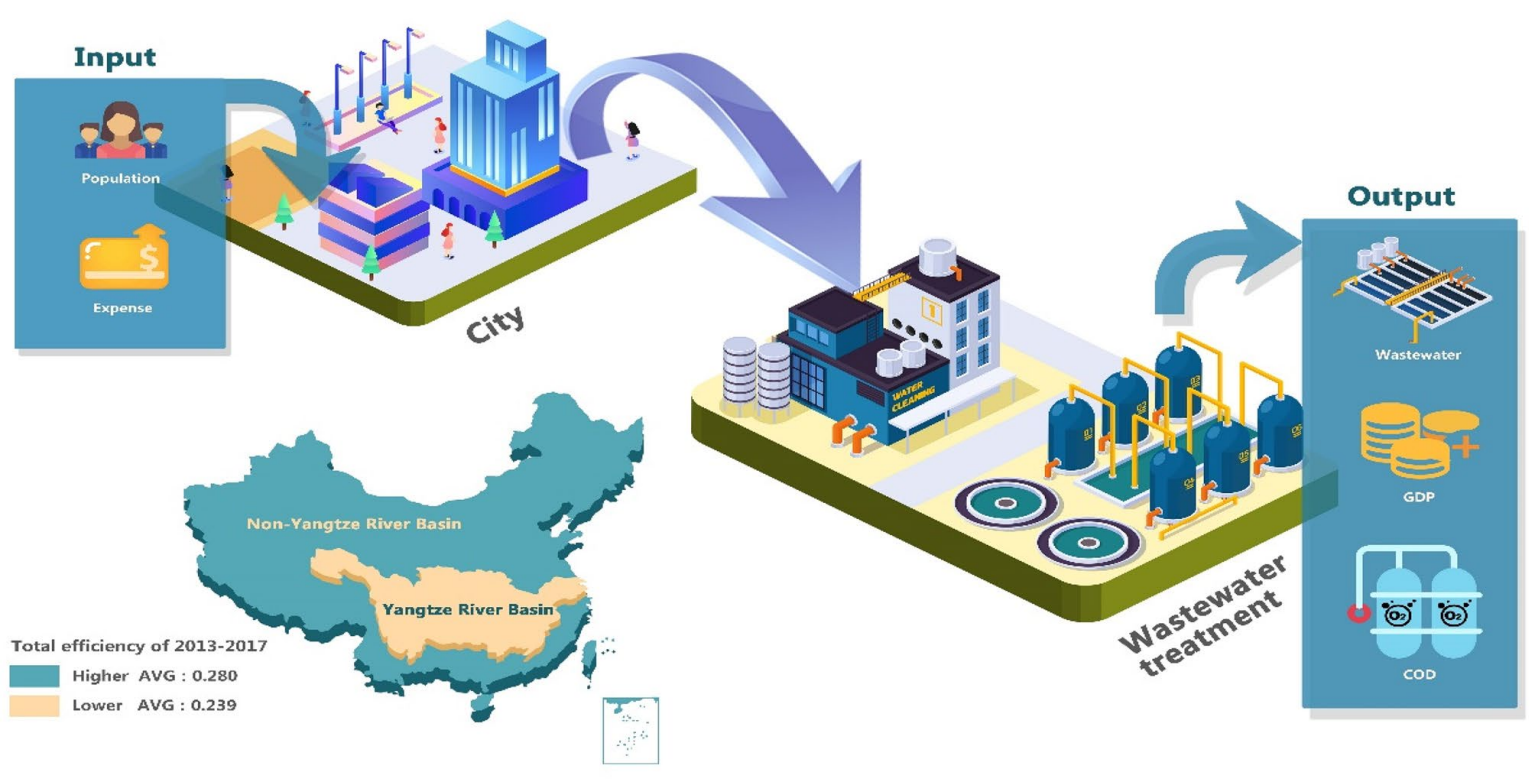
Input–output process of wastewater treatment between YRB and NYRB*. *The figure is created by the software of Adobe Illustrator (2020). https://www.adobe.com/cn/products/illustrator.html.

The remainder of this paper runs as follows. Section “Literature review” presents the literature review. Section “Research method” details wastewater treatment efficiency using a modified Dynamic DEA model as well as the dataset of inputs and outputs. Sections “Empirical results” and “Discussion” gives the empirical results and a discussion. Section “Conclusion and recommendation” offers conclusions and recommendations associated with wastewater treatment. Figure 1 illustrates the input–output process of wastewater treatment between YRB and NYRB.

## Literature review

Table 1 presents the current classification of studies on wastewater treatment efficiency.

1. Many scholars have studied the efficiency of industrial wastewater treatment, and conclusions have revealed that the current industrial water efficiency is low. Moreover, wastewater management and treatment efficiency as well as total factor efficiency (TFE) of wastewater control in industrial sectors are still far from optimal.
2. There are many studies on the wastewater treatment efficiency of wastewater treatment plants, and research on wastewater treatments plants have targeted various angles, such as greenhouse gas, the intensity of pollutants, the scale of plants, and so on. The construction of China’s wastewater treatment plants started late with low treatment technology and poor implementation of emission standards. Data distortions are also mentioned in studies of wastewater treatment efficiency of its wastewater treatment plants. Under the GDP-oriented appraisal system in China, the construction of wastewater treatment facilities is generally considered to be an important factor to promote GDP growth, and so local governments usually focus on the construction of wastewater treatment facilities, rather than the effective use of these facilities.
3. Many previous studies started from the time and spatial perspectives, but mainly focused on wastewater treatment efficiency in a basin in a province, or the efficiency of urban wastewater treatment at the prefecture-level city. Less attention has been paid to wastewater treatment along China’s important watersheds.

**Table 1:** Main research directions of this topic.

Aspect	Key indicators	Literature	Research methods	Main findings
Industrial water use/wastewater treatment efficiency	COD, NH_4_–N, water consumption	Wang et al.^10^	SBM-DEA; Shadow Price	There are still great potentials to reduce water consumption and pollutants’ discharge and great geographic disparities in different areas
	COD, ammonia nitrogen	Fujii and Managi^11^	WRDDM	The results indicate that wastewater management efficiency improved in the eastern and central regions. However, there is a significant efficiency gap between provinces in the western region
	Total energy consumption; industrial value added; industrial wastewater emissions	Yang and Li^12^	SBM-DEA and MATLAB programming	TFE of wastewater control in the industrial sectors is still far from optimal, and low wastewater control has become one of the obstacles to its sustainable development
	Industrial solid waste; assets	Ren et al.^13^	Dynamic modified SBM-DEA	In recent years the average efficiency of NYREB in many provinces shows a declining trend, and the average efficiency of solid waste treatment in provinces of YREB is mostly concentrated at a high level The total efficiency scores under the influence of urbanization are generally higher than that without the influence of urbanization level. Urbanization level has a significantly positive impact on wastewater output efficiency in each region Water pollution disease efficiency and the total efficiency of the eastern, western, and central regions all show a decreasing trend
	COD; urbanization rate water diseases; wastewater treatment capacity; COD	Sun et al.^14^ Sun et al.^15^	Dynamic exogenous variable SBM-DEA Dynamic network SBM-DEA	
Wastewater treatment plants	GHG; COD; climate type eco-efficiency; carbon footprint; CO_2;_ techno-economic efficiency; technological gap ratios; concentration of pollutants	Zhang et al.^16^; Dong et al.^17^; Gémar et al.^18^	WRDDM.; Combining DEA with uncertainty assessment	The operational costs and greenhouse gas emissions are the main drivers reducing eco-productivity WWTPs in eastern and western China significantly outperform those in the central region in terms of mean efficiency and performance stability
		An et al.^26^	ESDA model; super-efficiency DEA; Malmquist index	From 2011 to 2015, urban wastewater discharge showed a spatial agglomeration trend Both technological upgrade and scale-up efficiency are negative, leading to low overall efficiency
		Zhang et al.^27^	Statistical data analysis	Unbalanced population distribution and economic development led to differences in the efficiency of wastewater treatment plants between regions
		Hernández-Sancho et al.^28^; Hernández-Sancho et al.^29^	Non-radial DEA	The efficiency levels for the studied sample of WWTPs are low Plant size, quantity of eliminated organic matter, and bioreactor aeration type are significant variables affecting the energy efficiency of WWTPs
		Huang et al.^30^	Applied energy	The study evaluated the energy efficiency of wastewater treatment plants in the Yangtze River Delta and gave perspectives on regional discrepancies
		Jiang et al.^31^	SBM-DEA	Large WWTPs operate more efficiently than small ones. Of these, 170 wastewater treatment plants are relatively efficient, with a score of 1; 691 low-efficiency samples have different degrees of excess input or insufficient output
WU-WT system (water use and water treatment)	Water use; capital invested; wastewater treatment: wastewater discharge cost capital	Zhou et al.^32^; Hu et al.^33^	Mixed network two-stage SBM-DEA model;	In the past ten years, the WU efficiencies are often higher than the WT efficiencies The WT efficiencies are often lower than the WU efficiencies during 2006–2015
			Bi-level programming (BLP) and DEA	It is found that water systems can be cost-effective only when both water use and wastewater treatment subsystems are cost-effective
Urban wastewater treatment efficiency (UWWTE)	Length of sewage pipeline; daily treatment capacity; total amount of wastewater treated; dry sludge	Bian et al.^34^	Dynamic DEA	In China the main reason for the low efficiency of regional urban sewage purification systems is the poor sewage purification effect The results show that the overall UWTE is at a low level, as evidenced by the fact the average efficiency score is 0.51 during 2008–2017, and no cities have an efficiency score equal to 1 in the Yangtze River Economic Belt
		Pan et al.^35^	Bootstrap-DEA model and Malmquist index	
Agricultural water use efficiency	Number of agricultural workers; agricultural water consumption; agricultural fixed assets	Wang et al.^25^	SFA and spatial econometrics	AWUE of all provinces showed an upward trend during the observation period with obvious spatial correlation and unbalanced development of provinces
	Labor; capital water resources; agricultural production; greywater	Huang et al.^36^; Yang et al.^24^	Modified gravity model; SBM-DEA; social network analysis method; QAP	The overall trend of AWUE in China has been fluctuating and declining, and the structure of AWUE spatial network in China is complex and relatively stable with close inter-provincial connection and obvious spatial spillover effect. Geographical proximity, technological development level, farmers’ income, and natural resource endowment have a significant impact on the development of AWUE network

To sum up, the current research results in the international scope mainly are on the efficiency evaluation of wastewater treatment plants. However, due to regional differences, economic levels, and river crossings, China needs to target wastewater treatment in the areas along river basins. Thus, this study divides geographic area in China into the YRB and the NYRB and uses a modified Dynamic DEA model to evaluate and compare the wastewater discharge and treatment efficiencies of the two regions.

The main contributions of this paper are as follows. (1) Using fixed assets as a carry-over variable, the Dynamic DEA method is more scientific than the old static DEA method, as the former can better measure the change of wastewater treatment efficiency over time. (2) Compared with previous research results^37,38^, the combination of undesired output variables and Dynamic models improves the accuracy of efficiency assessment. (3) Perspective of YRB (a total of 18 provinces, including main streams and tributaries) and NYRB (a total of 12 provinces) is distinguished from previous studies’ geographical divisions of the eastern, central, and western regions^14,15^, bringing the research more in line with the background of green development and ecological protection of important river basins in China. Therefore, it urges China to attach importance to ecological construction along important rivers and promote rational, orderly and rapid development of river basins. (4) Taking 2018 as the cut-off point, two stages are divided into different stages of wastewater treatment so as to solve the problem and improve the accuracy of analysis and the degree of difference. Stage 1 is 2013–2017, which is the total amount control phase of wastewater treatment, with the focus on reducing the total amount of wastewater discharged. Stage 2 is 2018–2020, which is the pollution control phase of wastewater treatment, with the focus on reducing the amount of pollutants discharged from wastewater. The division of these two stages is of great significance for subdividing the spatial and temporal differences of wastewater treatment efficiency between YRB and NYRB.

## Research method

### A modified dynamic DEA model

The DEA method takes the weight of each input and output index as a variable and determines the optimal weight by solving the linear programming problem, so as to evaluate from the perspective of the most favorable evaluation unit while avoiding the influence of the evaluator’s subjective intention on the evaluation result when the weight is determined artificially.

**Figure 2:**
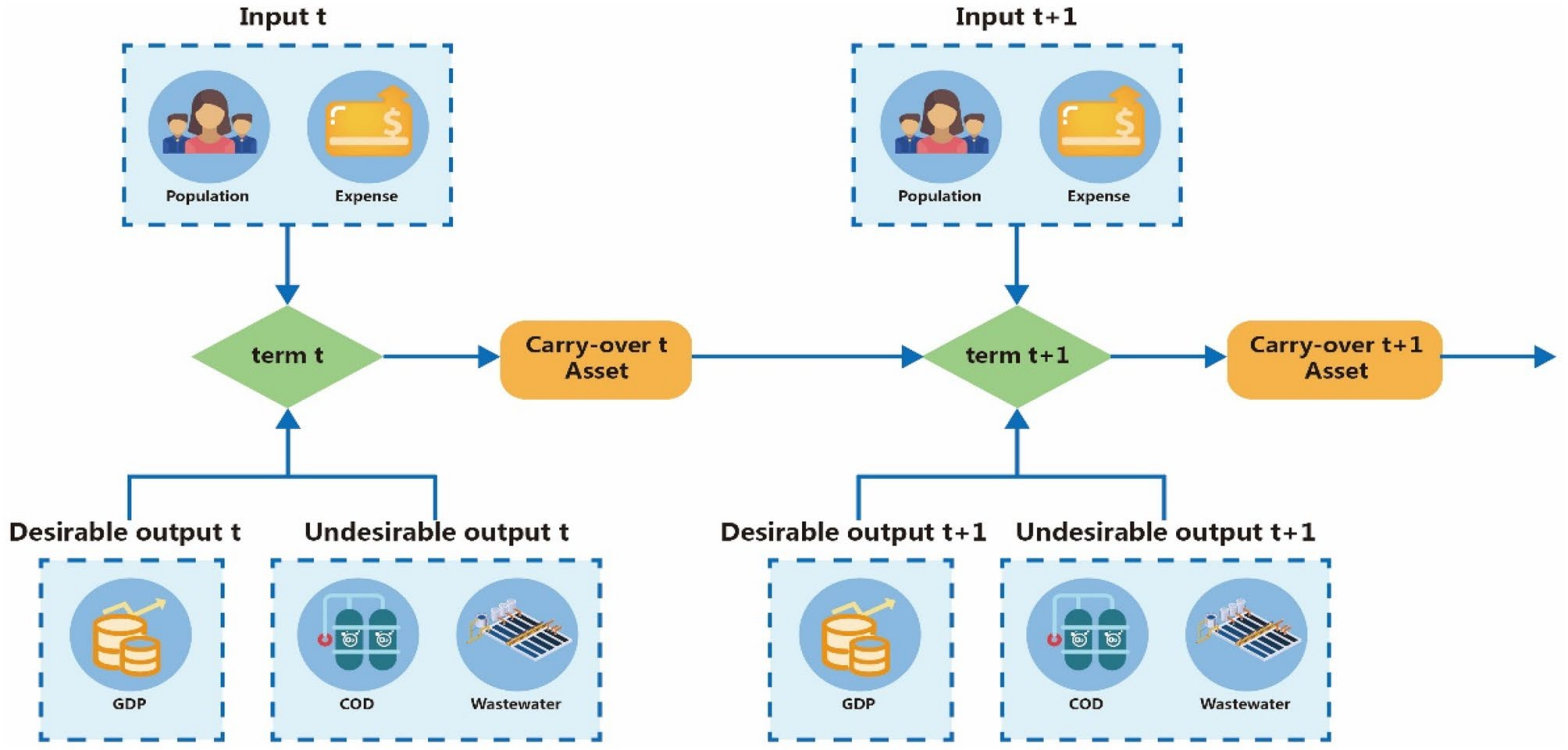
The structure of dynamic DEA.

Since this study considers undesirable output, the Tone and Tsutsui^39^ dynamic Slack-Based Measures (SBM) model can be modified to include undesirable output in the non-oriented Dynamic DEA model. Each period has independent input and output in every decision-making unit (DMU), and there is a carry-over link from periods *t* to *t* + *1* so as to find the change across two periods when inputs and outputs as classified as desirable and undesirable. Figure 2 shows the structure of the modified Dynamic DEA model.

This model sets up *n* DMUs *(j* = *1, 2, …, n*) over T periods (*t* = *1, 2, …, T*). The DMUs have multiple different and independent inputs and outputs in each term, with the *z* as a carry-over from period *t* to period *t* + *1* herein. Moreover, ${\lambda }_{j}$ connects the effective point to form the frontier. The carry-over *z* has four categories of good, bad, free, and fixed, which can be guaranteed by Eq. (1).

1 $${\sum }_{j=1}^{n}{z}_{ijt}^{\alpha }{\uplambda }_{j}^{t}={\sum }_{j=1}^{n}{z}_{ijt}^{\alpha }{\uplambda }_{j}^{t+1} \left(\forall \text{i};t=1,\dots ,T-1; \sum_{\text{j}=1}^{\text{n}}{\uplambda }_{\text{j}}^{\text{t}} =1 \right)$$

The non-oriented overall efficiency δ* with undesirable outputs in the modified Dynamic DEA is calculated by Eq. (2). Here, ${W}^{t}$ is the relative weight of the variable *t*. ${x}_{ijt} \left(\text{i}=1,\text{k},\text{m}\right)$ and ${Y}_{ijt} \left(\text{i}=1,\text{k},\text{s}\right)$ represent the inputs and outputs of the DMUs respectively. Y is divided into (${y}^{g}$, ${y}^{b}$), where ${y}^{g}$ is a desirable output, and ${y}^{b}$ is an undesirable output.

2 $$ {\updelta }^{*} = min\frac{{\frac{1}{T}\sum\nolimits_{t = 1}^{T} {w^{t} } \left[ {1 - \frac{1}{m}\sum\nolimits_{i = 1}^{m} {\frac{{s_{it}^{ - } }}{{x_{iot} }}} } \right]}}{{\frac{1}{T}\sum\nolimits_{t = 1}^{T} {W^{t} } \left[ {1 + \frac{1}{{s_{1} + s_{2} + ngood}}\left( {\sum\nolimits_{l = 1}^{{s_{1} }} {\frac{{s_{jt}^{ + g} }}{{y_{lot}^{g} }}} + \sum\nolimits_{l = 1}^{{s_{2} }} {\frac{{s_{jt}^{ - b} }}{{y_{lot}^{b} }}} + \sum\nolimits_{r = 1}^{ngood} {\frac{{s_{rt}^{good} }}{{z_{rot}^{good} }}} } \right)} \right]}}\quad \left( {{\text{i}} = 1, \ldots ,{\text{T}}} \right) $$

Equations (3)–(8) are the corresponding constraints.

3 $${x}_{\text{iot}}=\sum_{\text{i}=1}^{\text{m}}{\uplambda }_{\text{i}}^{\text{t}}+{\text{s}}_{\text{it}}^{-} \left(\text{i}=1,\dots ,\text{m};\text{t}=1,\dots ,\text{T}\right)$$

4 $${y}_{lot}=\sum_{l=1}^{s1}{y}_{lot}^{+g}{\lambda }_{j}^{t}-{s}_{lt}^{+g} \left(l=1,\dots ,s1;t=1,\dots ,T\right)$$

5 $${y}_{lot}=\sum_{l=1}^{s2}{y}_{lot}^{-b}{\lambda }_{j}^{t}-{s}_{lt}^{-b} \left(l=1,\dots ,s2;t=1,\dots ,T\right)$$

6 $$z_{{{\text{rot}}}}^{{{\text{good}}}} { = \sum\nolimits_{{{\text{r}} = 1}}^{{{\text{ngood}}}} {z_{{{\text{rot}}}}^{{{\text{good}}}} \lambda _{{\text{j}}}^{{\text{t}}}  - s_{{{\text{rt}}}}^{{{\text{good}}}} } \;\;} \left( {{\text{r}} = 1, \ldots ,{\text{ngood}};\;\;{\text{t}} = 1, \ldots {\text{T}}} \right)$$

7 $$\sum_{j=1}^{n}{\lambda }_{j}^{t}=1\left(t=1,\dots ,T\right), \sum_{t=1}^{T}{W}^{t}=T , \sum_{i=1}^{m}{W}_{i}^{-}=m$$

8 $${\lambda }_{j}^{t}\ge 0,{s}_{it}^{-}\ge 0,{s}_{lt}^{+g}\ge 0{,{s}_{lt}^{-b}\ge 0,s}_{rt}^{good}\ge 0.$$

The most efficient solution $\updelta$ is therefore in Eq. (9). And this study uses MaxDEA7.0 software for all data calculations.

9 $$\updelta =\frac{1-\frac{1}{m}\sum_{i=1}^{m}\frac{{s}_{iot}^{-*}}{{x}_{iot}}}{1+\frac{1}{{s}_{1}+{s}_{2}+ngood} \left(\sum_{l=1}^{s1}\frac{{s}_{jt}^{+g*}}{{y}_{lot}}+\sum_{l=1}^{s2}\frac{{s}_{jt}^{-b*}}{{y}_{lot}}+\sum_{r=1}^{ngood}\frac{{s}_{rot}^{good*}}{{z}_{rot}^{good}} \right)} \left(\text{i}=1,\dots ,\text{T}\right)$$

### Data and variables

This paper employs panel data from 30 provinces and cities in China in 2013–2020, which cover YRB (a total of 18 provinces and cities, including the main stream and tributaries; Anhui, Chongqing, Guizhou, Hubei, Hunan, Jiangsu, Jiangxi, Shanghai, Sichuan, Yunnan, Zhejiang, Fujian, Gansu, Henan, Shaanxi, Guangdong, Guangxi, and Qinghai) and NYRB (a total of 12 provinces and cities; Beijing, Hainan, Hebei, Heilongjiang, Jilin, Liaoning, Inner Mongolia, Ningxia, Shandong, Shanxi, Tianjin, and Xinjiang). Tibet is excluded because the data are hard to come by, and its wastewater treatment facilities and methods cannot be compared with those of other regions. Data from the years 2013 to 2020 are collected from the Statistical Yearbook of China, China Environmental and Protection Bureau reports, and China Energy Statistics Yearbook. Variables of this study are in the following Table 2.

**Table 2:** Input and output variables.

Input variables	Desirable output variable	Undesirable output variables	Carry-over variable
Population Expense	GDP	Wastewater COD	Assets

#### Input variables

##### Population

The number of permanent residents at midnight on December 31 of each region. Unit: 10,000 people.

##### Expense

The total amount of input costs for wastewater treatment in each region. Unit: RMB 100 million.

#### Desirable output variable

##### GDP

The final result of production activities of all units in each region at a market price for a certain period of time, reflecting the economic strength of the region. Unit: RMB 100 million.

#### Undesirable output variables

##### Wastewater

The sum of wastewater discharge and domestic sewage discharge in the region. Unit: 10,000 tons.

##### COD

The sum of COD emissions in wastewater and COD emissions from domestic sewage, used to indicate the content of organic matter in wastewater, reflecting the degree of organic pollution in water. The higher the COD value is, the heavier are the organic pollutants in the water. Unit: 10,000 tons.

#### Carry-over variable

##### Assets

A general term for the amount of work and the costs associated with the construction and acquisition of fixed assets in a given period of time in the form of money. This is a comprehensive indicator reflecting the scale, structure, and development speed of fixed asset investment and an important basis for observing the progress of a project and assessing the investment effect. Unit: RMB 100 million.

### Statistical analysis of input–output indicators

**Figure 3:**
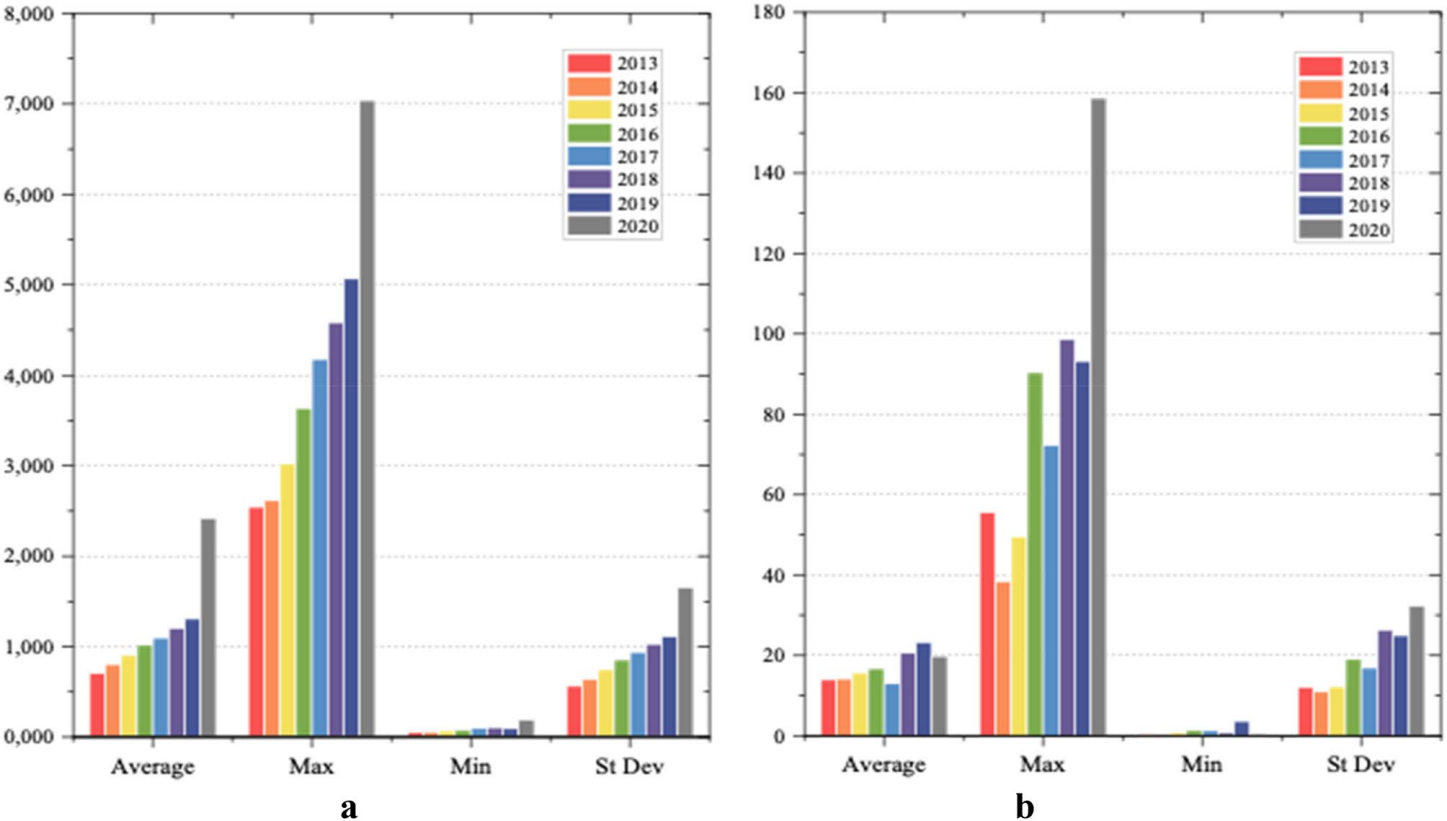
Statistical analysis of input indicators in YRB and NYRB from 2013 to 2017. (**a**) Population (input), (**b**) Expense (input).

**Figure 4:**
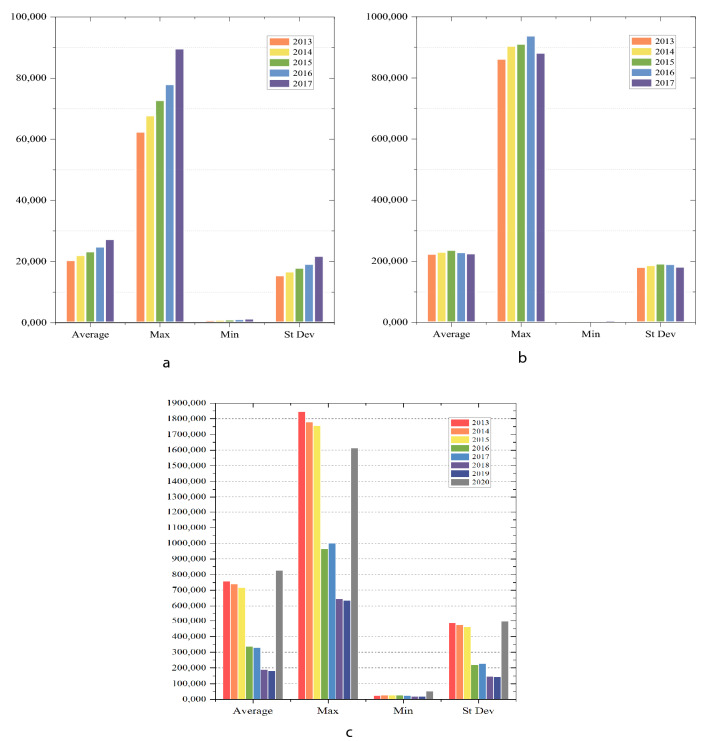
Statistical analysis of output indicators in YRB and NYRB in Stage 1. (**a**) GDP (output), (**b**) Wastewater (output), (**c**) COD (output).

Figures 3 and 4 shows the statistical illustration of input and output variables by years. It can be seen from Fig. 3a that the maximum population has grown steadily over the eight years, and the standard deviation trend has not shown significant fluctuations, indicating that the population development level of each region is relatively balanced. The average population has increased slightly, and the overall level has remained basically stable.

Figure 3b presents that the maximum value of wastewater treatment cost investment fluctuates greatly within the eight years. The maximum value of wastewater treatment expenses in 2015–2016 has increased sharply, indicating that the input costs of some provinces and cities have soared. Overall, the average value of wastewater treatment input costs increased steadily between 2013 and 2016, and there is a clear decline in 2016–2017. However, the maximum value of wastewater treatment expenses in 2020 exceeds 150, while the average value shows a decrease.

It can be seen from Fig. 4a that the average, maximum, minimum, and standard deviation values of GDP have increased over a period of eight years. This shows that the GDP levels of all provinces and cities in the country have maintained a steady increase. Figure 4b shows that the average value of wastewater discharge did not change significantly, but the maximum value increased steadily from 2013 to 2016 and fell slightly in 2017. The difference between the minimum and maximum values is large, and the minimum has been kept to a small increase. It means that the overall national wastewater discharge is still growing in volatility. Figure 4c shows that the average, maximum, minimum, and standard deviation values of COD content in wastewater from 2013 to 2015 have remained at a high level, but the indicators in 2016 have shown a huge decline.

## Empirical results

The empirical analysis is divided into two stages for discussion. Stage 1 is 2013–2017, which is the total amount control phase of wastewater treatment, with the focus on reducing the total amount of wastewater discharged. Stage 2 is 2018–2020, which is the pollution control phase of wastewater treatment, with the focus on reducing the amount of pollutants discharged from wastewater.

Among the total DMU, cities and autonomous regions in Table 4 (Appendix A), the five-year average efficiency score is 0.902, 0.398 for the YRB and 0.504 for the non-YRB. There are three cities with the optimal efficiency over the five-year period, namely Beijing, Shanghai, and Tianjin, of which Shanghai belongs to YRB. The total efficiency scores of the remaining 27 provinces, cities and autonomous regions are all less than 1, accounting for 90% of the total number of DMUs, indicating that these regions’ efficiency needs to be improved. Among the remaining 27 provinces, Liaoning has the highest five-year average score of 0.794, while Gansu has the lowest score of 0.252. Between 2013 and 2017, Liaoning has achieved optimal efficiency three times. The five-year average efficiency value of each province in YRB is 0.398 while the average efficiency value of the NYRB is 0.504, which is better than YRB.

### Ranking of total wastewater treatment efficiency in Stage 1

Table 3 shows the rankings of total efficiency scores for each province in Stage 1. Beijing, Shanghai, Tianjin, Liaoning, Jiangsu, Guangdong, Zhejiang Shandong, Fujian and Inner Mongolia rank in the top ten in terms of efficiency values. Among them, Shanghai, Jiangsu, Guangdong, Zhejiang and Fujian belong to YRB, while Beijing, Tianjin, Liaoning Shandong and Inner Mongolia belong to NYRB.

**Table 3:** Total efficiency scores and rankings of provinces from 2013 to 2017.

No.	DMU	2013		2014		2015		2016		2017		AVE (5 years)
		Score	Rank	Score	Rank	Score	Rank	Score	Rank	Score	Rank	
1	Anhui	0.3172	25	0.2361	28	0.2923	26	0.2742	21	0.3035	21	0.2847
2	Zhejiang	0.4949	6	0.479	7	0.5306	9	0.4709	8	0.51	7	0.4971
3	Chongqing	0.38	13	0.3634	11	0.725	5	0.3517	14	0.3775	15	0.4395
4	Fujian	0.4096	10	0.3894	9	0.4744	11	0.4565	10	0.5042	8	0.4468
5	Gansu	0.3083	28	0.2144	31	0.2771	28	0.222	31	0.2379	29	0.2519
6	Guangdong	0.5235	4	0.5204	5	0.5635	8	0.4333	11	0.4524	11	0.4986
7	Guangxi	0.3144	27	0.2597	24	0.3257	24	0.4574	9	0.4624	10	0.3639
8	Guizhou	0.3059	30	0.2331	29	0.282	27	0.2829	20	0.2907	23	0.2789
9	Sichuan	0.3315	20	0.2797	20	0.4274	14	0.2461	27	0.2677	25	0.3105
10	Shaanxi	0.3929	12	0.3005	15	0.3885	18	0.3373	17	0.3681	17	0.3575
11	Shanghai	1	1	1	1	1	1	1	1	1	1	1
12	Henan	0.3341	19	0.2849	18	0.4064	17	0.3499	15	0.3848	14	0.352
13	Hubei	0.3506	16	0.319	13	0.3838	21	0.3448	16	0.3724	16	0.3541
14	Hunan	0.3304	21	0.3055	14	0.3527	23	0.3112	19	0.3325	19	0.3265
15	Yunnan	0.3066	29	0.2499	25	0.3883	19	0.2421	28	0.2589	26	0.2892
16	Jiangsu	0.5032	5	0.5214	4	0.6298	6	0.5396	5	0.6019	5	0.5592
17	Jiangxi	0.3147	26	0.2488	26	0.3096	25	0.2578	24	0.294	22	0.285
18	Qinghai	0.3413	17	0.2316	30	0.2699	29	0.2275	30	0.2375	30	0.2616
YRB AVE score		0.4033		0.3576		0.4459		0.3781		0.4031		0.3976
19	Ningxia	0.3257	23	0.2966	16	0.4352	12	0.2509	25	0.2797	24	0.3176
20	Hebei	0.3566	15	0.287	17	0.4872	10	0.3165	18	0.3504	18	0.3595
21	Jilin	0.3985	11	0.321	12	0.385	20	0.4317	12	0.4141	13	0.3901
22	Shandong	0.4183	9	0.4069	8	0.599	7	0.5066	6	0.533	6	0.4928
23	Heilongjiang	0.3353	18	0.2757	22	0.4338	13	0.3653	13	0.4249	12	0.367
24	Shanxi	0.3651	14	0.2447	27	0.2682	30	0.2633	23	0.3169	20	0.2916
25	Inner Mongolia	0.4728	7	0.3697	10	0.4212	15	0.4717	7	0.4768	9	0.4424
26	Tianjin	1	1	1	1	1	1	1	1	1	1	1
27	Xinjiang	0.3254	24	0.2633	23	0.367	22	0.2495	26	0.2543	27	0.2919
28	Beijing	1	1	1	1	1	1	1	1	1	1	1
29	Hainan	0.3281	22	0.2769	21	0.4105	16	0.2374	29	0.2522	28	0.301
30	Liaoning	0.4636	8	0.5041	6	1	1	1	1	1	1	0.7935
NYRB AVE score		0.4825		0.4372		0.5673		0.5077		0.5252		0.5040

In YRB, the rankings of eight provinces have risen in the 5 years, the rankings of seven provinces have declined. The rankings of Jiangsu and Shanghai have been relatively stable. The provinces with significant increases in efficiency and rankings are Anhui, Guangxi, Henan, Jiangxi, Guizhou, and Yunnan. Among them, Guangxi rose the most, up 17 places, and its efficiency value increased from 0.314 in 2013 to 0.462 in 2017. Second, Guizhou increased by 7 places. It shows that the wastewater treatment measures in these provinces and cities are powerful and that efficiency has obviously improved.

Qinghai is one province with a severe drop in rankings. In 2013, its efficiency value was 0.341, ranking 17th, and by 2017 it fell to 0.238 or 30st. Therefore, this province must strengthen wastewater discharge management, should not neglect wastewater treatment due to excessive pursuit of economic development, and seek appropriate treatment programs to improve wastewater treatment efficiency. Zhejiang, Gansu, Shaanxi, Sichuan, and other provinces have also decreased in rankings and thus should be more aware of their situation.

In NYRB the rankings of three provinces, municipalities, and autonomous regions have risen within five years, while the rankings of six have declined. Those with rising rankings are Liaoning, Shandong, and Heilongjiang. The largest increase is by Heilongjiang, whose efficiency value increased from 0.335 in 2013 to 0.425 in 2017, and its ranking rose by 6. The efficiency score of Liaoning rose from 0.464 to 1, indicating that it has achieved the best efficiency through years of efforts. Among NYRB, the decline in Shanxi is more obvious. In 2017, its ranking dropped 5 places compared with 2013. On the whole, Shanxi ranked in the middle and upper reaches in 2013, but dropped significantly to 30st in 2015. This situation needs to be paid greater attention in order to prevent the deterioration of wastewater treatment conditions.

Although the rankings of Xinjiang and Ningxia have not dropped significantly, they have low efficiency values and are lagging behind. They exhibit no significant improvements for total efficiency after five years’ governance. For example, Ningxia’s ranking in 2013–2015 rose significantly and then fell back to the original level. Therefore, these two need to take appropriate measures with precision and force and continue to do a good job in wastewater treatment. The rankings of other provinces in the region slightly fluctuate within a reasonable range.

After comparing the changes in the total efficiency scores within YRB and NYRB, most provinces in NYRB rank better than those of YRB. In addition, the efficiency of wastewater treatment in economically developed provinces is better than other provinces, and wastewater treatment in central and western provinces still needs continuous improvement.

### Wastewater and COD discharge efficiency scores and rankings of provinces in Stage 1

**Figure 5:**
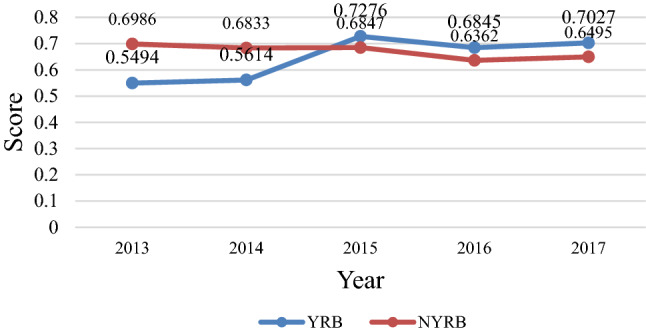
Average level of wastewater discharge efficiency scores in YRB and NYRB in Stage 1.

**Figure 6:**
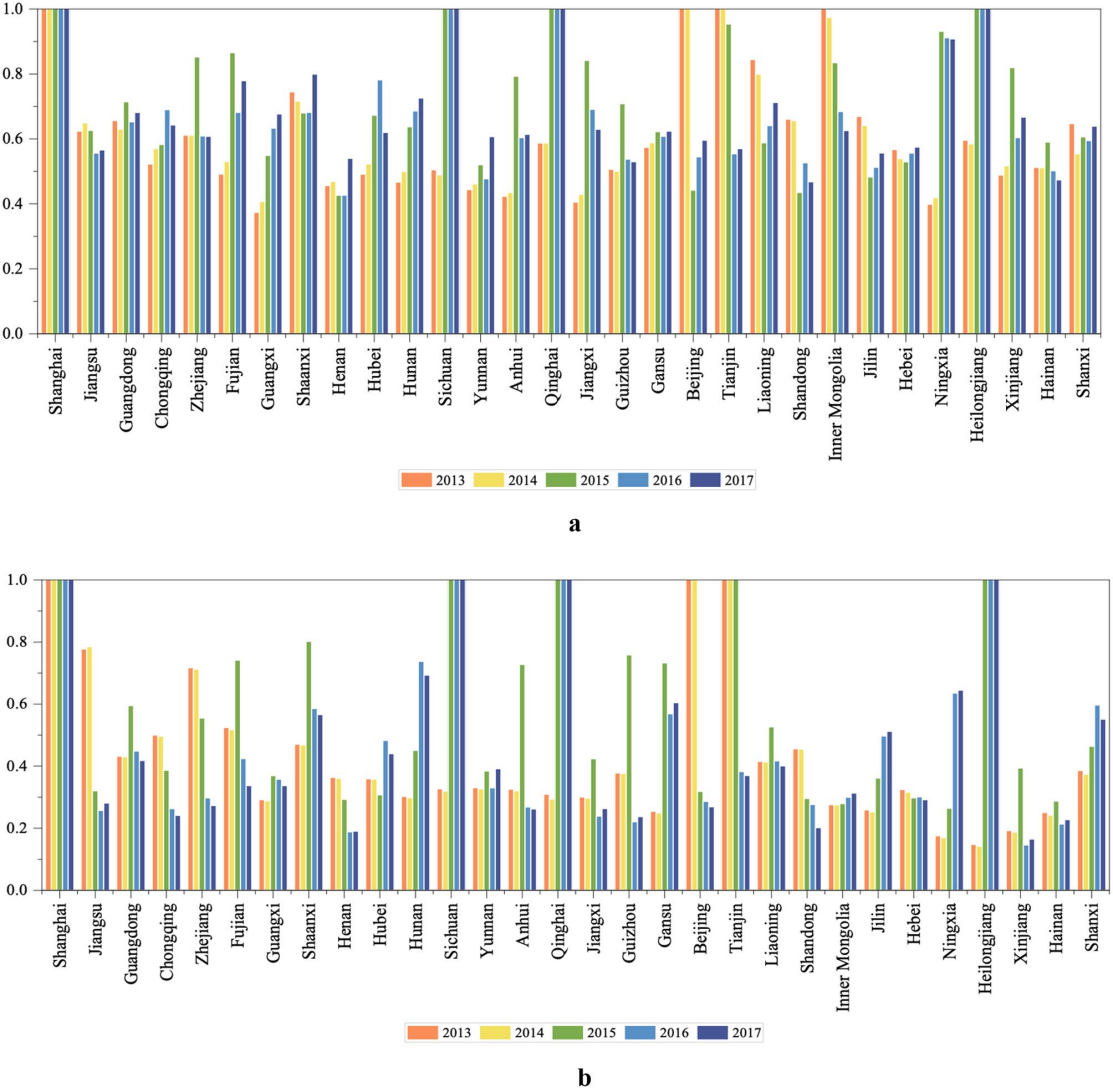
Changes in undesirable output variables efficiency values in Stage 1. (**a**) Changes in the efficiency values of wastewater discharge from 2013–2017, (**b**) Changes in the efficiency values of COD discharge from 2013–2017.

It can be seen from Fig. 5 that the fluctuation rate of wastewater discharge efficiency in the two regions is relatively flat during the five years. Moreover, the wastewater discharge efficiency value of NYRB has slightly decreased, while that of YRB has increased significantly in 2014–2015. It is worth noting that Shanghai’s wastewater discharge and COD efficiency values are both optimal, and there is also a large gap between the wastewater discharge and COD efficiency scores of some provinces from Figs. 5 and 6.

The wastewater discharge efficiency values of most provinces in YRB are increasing. The provinces with obvious improvement are Fujian, Guangxi, Hubei, Hunan, Sichuan, Qinghai, and Jiangxi; in fact, the wastewater discharge efficiency values of Sichuan and Qinghai have been 1 since 2015. This indicates that their wastewater discharge has improved through efforts to achieve an effective state. The efficiency values of the two provinces of Jiangsu and Zhejiang have declined slightly and need to be addressed.

In NYRB the wastewater discharge efficiency values of Ningxia, Heilongjiang, and Xinjiang have increased, with the first two gaining significantly. Heilongjiang rose from 0.596 to 1 in 2015 and remained stable afterwards; Ningxia rose from 0.399 to 0.908 and is expected to reach 1 through continuous efforts. Beijing, Tianjin, and Inner Mongolia and other provinces have seen significant declines in wastewater discharge efficiency. The efficiency value of Inner Mongolia decreased from 1 in 2013 to a medium level around 0.6 in 2017. In Beijing and Tianjin, their total efficiency values scored at 1 for 5 consecutive years, while their wastewater discharge efficiency has continued to decline, indicating that management of wastewater discharge has been neglected and needs to be taken seriously.

Comparing the wastewater discharge efficiency scores of YRB and NYRB, we find that the wastewater discharge efficiency values of YRB in most areas in 2013 are less than 0.5, but went above 0.5 × 2015. Shanghai has maintained an efficiency score of 1 for 5 consecutive years. In most provinces other than YRB, there has been a slight decline. Therefore, the wastewater discharge efficiency of YRB is generally better than that of NYRB.

The COD efficiency scores of 8 provinces in YRB have increased significantly, including Guangxi, Shaanxi, Hunan, Hubei, Sichuan, Yunnan, Qinghai, and Gansu, with Sichuan and Qinghai having the largest increase of reaching 1 in 2015 and staying there afterwards. The COD efficiency values of the remaining 10 provinces have declined in varying degrees, with Jiangsu and Zhejiang showing the largest drops. Jiangsu fell from 0.778 to 0.281, and Zhejiang fell from 0.717 to 0.274. The COD efficiency scores of these two provinces have been declining year after year, and wastewater discharge efficiency has always been in the middle level of 0.6, indicating that while the economy is developing, the control of wastewater treatment is not strict, and efforts should be made to improve the efficiency of wastewater discharge and pollution treatment.

The wastewater discharge efficiencies in Guangdong, Chongqing, Henan, Fujian, Anhui, Jiangxi, Guizhou and other provinces increased slightly in five years, but their COD efficiency values are very low. For example, the COD efficiency value in Henan dropped from 0.364 to 0.191, indicating the area should not only control the total amount of wastewater discharge, but also pay attention to the related treatment of wastewater pollution.

Figure 6 shows the changes in wastewater discharge and COD efficiency values of YRB and NYRB in 2013–2017. Comparing Fig. 6a,b, we see that the COD efficiency value of China’s wastewater is lower than the wastewater discharge efficiency value. In response to the increasingly serious problem of water pollution in China, the central government introduced and implemented the new Environmental Protection Law and the Water Pollution Prevention Action Plan in 2015. The implementation of these laws and regulations has achieved mixed results in wastewater discharge and COD governance in various provinces. The specific analysis runs as follows.

In 2015 the wastewater discharge and COD efficiency values of Guangdong, Fujian, Sichuan, Anhui, Qinghai, Jiangxi, Guizhou, Heilongjiang, and other provinces increased significantly at the same time. Among them, Guangdong, Fujian, Sichuan, Anhui, Qinghai, and Guizhou belong to YRB. Although the COD efficiency values of Shaanxi and Liaoning increased significantly in 2015, their wastewater efficiency value fell. The wastewater efficiency value of Zhejiang increased significantly in 2015, but its COD efficiency value dropped significantly. It shows that Zhejiang has put its focus on wastewater discharge during the year, and the treatment of COD content in wastewater has been relaxed. In addition, some provinces have experienced significant declines in wastewater discharge and COD efficiency values after 2015, and some have even dropped to lower levels, indicating that wastewater pollution and treatment work is very complicated and should always be unremitting.

### Analysis of wastewater treatment expense in provinces in Stage 1

**Figure 7:**
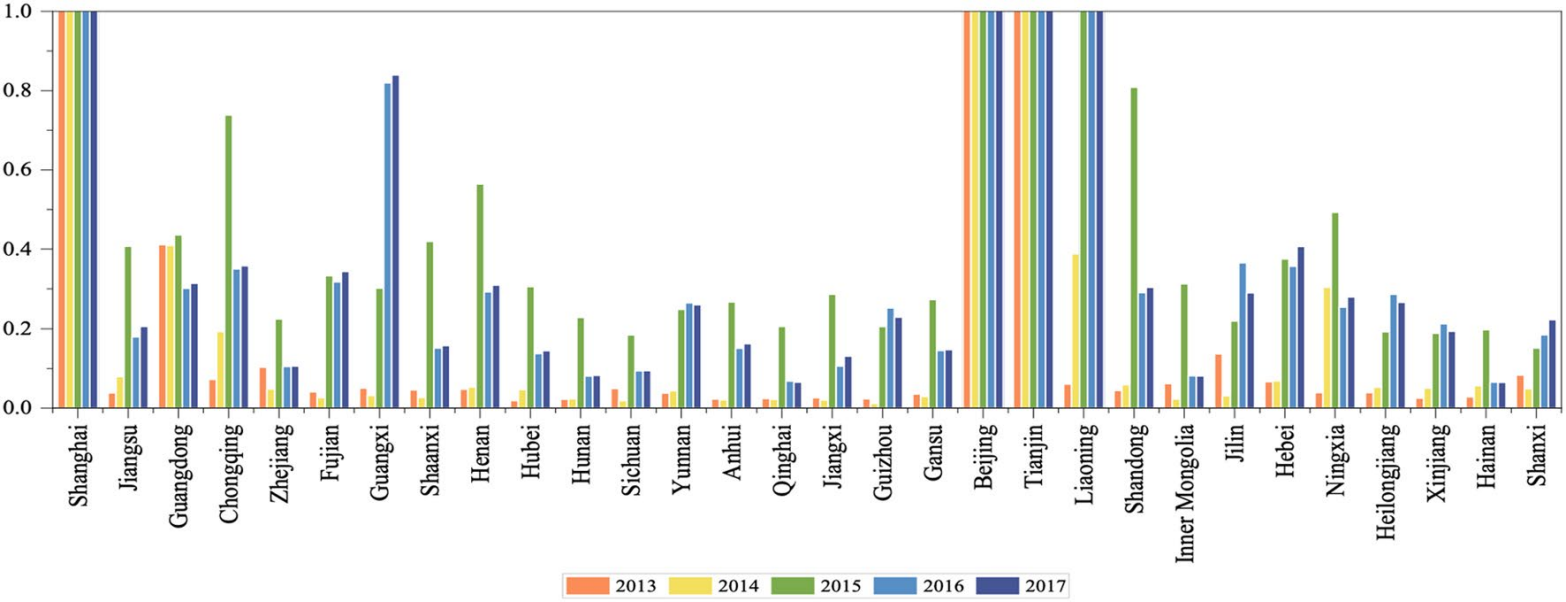
Changes in the efficiency value of wastewater treatment expense from 2013 to 2017.

Through Fig. 7 and Table 5 (Appendix B), we confirm a large gap in the cost efficiency of wastewater treatment in various provinces. Shanghai, Beijing, and Tianjin maintained an optimal efficiency value of 1 for five consecutive years. However, in 2013 the efficiency of wastewater treatment expense in 25 provinces of China was less than 0.1, which means a state of extremely low efficiency.

In YRB the efficiency of wastewater treatment input has been increasing continuously and Guangxi’s governance expense efficiency value that continued to increase, from 0.032 in 2014 to 0.840 in 2017 for a growth rate of 2491%, making it the province with the largest growth. Although wastewater discharge and COD treatment efficiency in Guangxi have also increased correspondingly, its growth rate is far lower than the growth rate of governance cost input efficiency, indicating that its administration cost allocation structure needs to be further adjusted and improved. Similar to the COD efficiency value, most provincial and municipal governance cost input efficiencies in YRB in 2015 showed a significant increase, but fell again in 2016–2017. We point out that the wastewater discharge efficiency and COD treatment efficiency of Sichuan and Qinghai have greatly improved to the optimal value of 1, but their treatment expense efficiency value is low. Thus, it is necessary to pay attention to the planning and use of wastewater treatment funds and to improve the efficiency of input costs.

In NYRB the efficiency of Liaoning’s treatment costs increased the most, but its wastewater discharge and COD efficiency values declined in five years. Similar circumstances appear for Beijing and Tianjin, whose governance expense efficiency scores have remained at 1 for five consecutive years, while their wastewater discharge efficiency and COD efficiency values have continued to decline. This means their governance cost allocation structure needs to be improved and optimized.

### Analysis of the current situation of China’s pollution emissions in Stage 2

**Figure 8:**
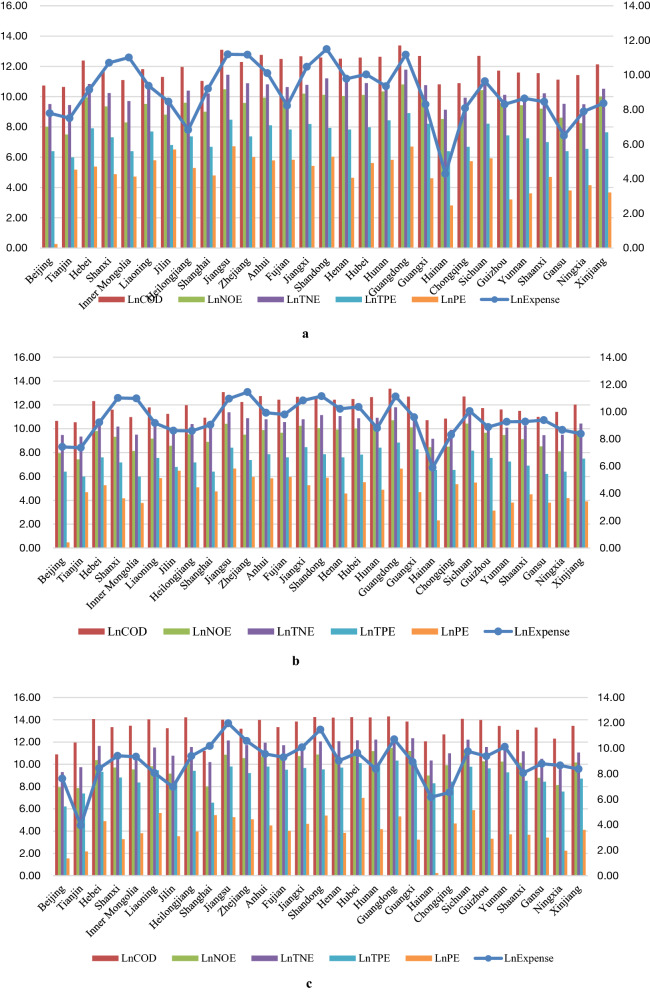
Natural log values of COD, NOE, TNE, TPE, and PE from 29 provinces in China in Stage 2*. *Source: Compiled by the authors themselves based on the data collected. In order to avoid absolute differences among the indicators and the influence of individual extreme values, we process the natural logarithm of the indicator values. The comparison between different variables and the same variable is also facilitated by this method, and the raw data are detailed in the “Supplementary material”. (**a**) Natural log values of COD, NOE, TNE, TPE, and PE from 29 provinces in China in 2018. (**b**) Natural log values of COD, NOE, TNE, TPE, and PE from 29 provinces in China in 2019. (**c**) Natural log values of COD, NOE, TNE, TPE, and PE from 29 provinces in China in 2020.

From Fig. 8a–c, we present the following results.

1. The level of economic development is one of the important drivers of pollutant emissions such as COD and nitrogen and oxygen, and economically developed provinces invest a large amount of money as wastewater treatment every year. The fluctuation of wastewater treatment in each province Fig. 8a is much larger than that of Fig. 8b,c. In terms of the total amount, 29 provinces show an inverted V-shape trend of wastewater treatment investment in Stage 2, where the investment in 2018, 2019, and 2020 is respectively 6.22 billion yuan, 6.95 billion yuan, and 5.74 billion yuan. Among them, Jiangsu ranks first in 2020 with wastewater treatment investment of 158.67 million yuan. Hainan’s wastewater treatment investment has been at a low level, but its pollutant emission is relatively high. With reference to the former part of the efficiency ranking, Hainan still needs to increase wastewater treatment investment to improve the overall wastewater treatment level. As a municipality directly under the central government, Tianjin has three consecutive decreases in wastewater treatment investment in these three years, from 18.19 million yuan in 2018 down to 520 thousand yuan in 2020, or a decrease of more than 95%.
2. From the perspective of pollutant emissions, COD and nitrogen emissions are still the key objects of pollution reduction and emission reduction. Among the pollutants counted, the emissions of COD are the largest, rising from 5.84 million tons in 2018 to 2.53 million tons in 2020, or an increase of 328.46%, of which Jiangsu is ranked first with 1.21 million tons of COD emissions. In addition, the emissions of total nitrogen should not be ignored, going from 1.91 million tons in 2018 to 3.19 million tons in 2020, and the emissions in 2020 are the sum of the emissions in 2018 and 2019. It is also worth noting that the emissions of petroleum continue to decline, with a decrease of 47.95%.
3. From YRB and NYRB, the gap between the two regions in provincial average wastewater treatment investment has gradually expanded. In 2020, the provincial average wastewater treatment investment in YRB and NYRB was 24.71 million yuan and 1.28 billion yuan respectively. In terms of pollutant discharge, the provincial average COD discharge in YRB rose from 253,500 tons in 2018 to 958,600 tons in 2020, or up more than 250%. NYRB also shows the same characteristics, moving up more than 450%. However, the provincial average discharge of petroleum in YRB and NYRB is decreasing, with YRB falling from 281.28 tons in 2018 to 167.59 tons in 2020, or a decrease of 40%. NYRB’s average provincial emissions of petroleum category in 2018 decreased from 197.66 tons to 72.89 in 2020, or a decrease of 60%.

## Discussion

1. First, previous studies have tended to divide China according to administrative divisions or geographical locations, such as Shi et al. divided China into eastern, central, and western regions and analyzed the efficiency of thirty provinces in both economic production and sewage treatment stages^40^. Tang et al. analyzed the differences in solid waste disposal efficiency between regions according to coastal and inland divisions^41^. Based on the characteristics of large differences among different river basins in China, this paper not only analyzes the efficiency of wastewater treatment of 30 provinces in eastern, central and western regions, but also analyzes the efficiency of wastewater treatment in each province from the perspective of YRB and NYRB, which is in line with the current emphasis on ecological and environmental management in Yangtze River basin in China. Combining the empirical results section, it can be found that the 5-year average efficiency in YRB is lower than that in the NYRB during the study period.
2. Second, since China no longer publishes the total wastewater discharge after 2018, this study adds the statistical analysis of Stage 2 and comparatively analyzes the discharge of pollutants in wastewater in each province between 2018 and 2020, which shows that the COD and total nitrogen emissions in wastewater do not show a decreasing trend, but the wastewater treatment investment shows a decreasing trend. The total COD emissions in YRB rose 250% and NYRB rose 450% compared with 2018. Although total wastewater emissions are no longer published, wastewater treatment can still be studied in the future from the perspective of different pollutants.
3. Third, in further studies, the efficiency of wastewater treatment can be studied considering different driving factors such as economic development level, environmental regulation, and city size. The efficiency of wastewater treatment in China can also be evaluated from the perspective of the implementation of policies such as “10-point Water Plan”, “River Chief Policy” and “Water Emissions Trading policy”.

## Conclusion and recommendation

### Conclusion

This study divided 30 provinces in China into YRB and NYRB, and the study period is divided into two phases, with the first phase being the total wastewater control phase and the second phase being the analysis of the pollutant control phase. The wastewater treatment efficiency from 2013 to 2020 is compared as well as regional differences and potentiality for improvement, so as to clarify the impact, efficiency factors, and improvement directions of wastewater treatment in each region.

1. From the perspective of total efficiency scores and rankings, the total efficiency values of various provinces in YRB and NYRB are generally low. In the total efficiency score of wastewater treatment, NYRB is 0.5040, which is slightly higher than that of YRB at 0.398, but the inter-provincial gap in NYRB is larger than that of YRB.
2. From the perspective of wastewater discharge efficiency, the wastewater discharge efficiency of NYRB is slightly better than that of YRB, where the scores of NYRB and YRB are 0.671 and 0.663, respectively, but the advantages are not obvious.
3. The efficiency value of COD is lower than the wastewater discharge efficiency value, but the overall change trend is consistent. In YRB and NYRB, provinces with COD efficiency values below 0.1 both account for 50%. The phenomenon of polarization between provinces is more serious.
4. The efficiency of governance expense is most consistent with the efficiency trend of COD, indicating that governance costs are the most direct input factors affecting wastewater treatment efficiency. Hence, usage planning and adjustment of governance expense should be given due attention
5. In terms of total wastewater treatment costs for emissions and inputs, the wastewater treatment inputs in Stage 2 show a decreasing trend, but the emissions of pollutants show an overall increasing trend, especially COD and total nitrogen emissions. Therefore, the key to wastewater treatment is still to promote industrial transformation, change the previous manufacturing development model, focus on industrial clusters and intelligent manufacturing, vigorously promote industrial digitalization and digital industrialization, and realize the sustainable development of industry, ecology, and environment.

### Recommendation

Based on the above research results and combined with the actual situations of YRB and NYRB, this research proposes the following countermeasures for the coordinated management of wastewater and the improvement of environmental efficiency in various provinces.

1. In view of the reality that wastewater discharge efficiency and COD efficiency are low overall, it is necessary to further explore the high-tech fields of wastewater treatment, appropriately treat and reuse wastewater of different water quality, and find new processes and equipment with high efficiency and low cost. Enterprises and research institutes with good professional and technical bases and strong R&D capabilities should help establish as soon as possible wastewater treatment technology and reuse engineering centers in various regions.
  In YRB, such as Sichuan, Qinghai, which have high efficiency of wastewater discharge and COD efficiency but low efficiency of treatment expense, relevant economic policies that encourage wastewater treatment should be executed to form a price formation mechanism for the sustainable development of the wastewater treatment industry. When introducing new technologies and new equipment, it is necessary to fully consider the actual situation in the local area. Blindly introducing new technologies and thus wasting resource and funds should not be allowed. Some economic means must be adopted to encourage wastewater reuse, and appropriate incentives or subsidies need to be given to wastewater treatment plants and enterprises that develop wastewater reuse.
2. In NYRB, such as Beijing and Tianjin where input and output efficiencies have dropped significantly, it is reasonable to allocate input costs, increase investment in wastewater treatment technology, improve the mechanization and automation of wastewater treatment, and strengthen training for managers and technicians. Water pollution treatment can be included in the industrial zone infrastructure construction planning and wastewater treatment planning, and wastewater and water informatization work can be improved.
3. For provinces such as Zhejiang, Inner Mongolia, and Xinjiang where wastewater treatment efficiency and wastewater discharge efficiency are extremely low, and for provinces such as Henan, Beijing, Tianjin, and Hainan where wastewater discharge efficiency continues to decline, the government needs to raise its sense of responsibility, strengthen its emphasis on wastewater treatment, formulate relevant policies for wastewater treatment, and set up special funds. The government can establish a comprehensive governance mechanism, strictly manage factories in accordance with relevant national wastewater discharge standards, ensure the enforceability of sewage discharge legislation, and severely punish any violations of laws and regulations.

## Supplementary Information

Supplementary Tables.

## Data Availability

All data generated or analyzed during this study are included in this published article.

## References

[1] Keiser DA, Shapiro JS (2018). Consequences of the clean water act and the demand for water quality*. Q. J. Econ..

[2] Santana, C. S. *et al*. Assessment of water resources pollution associated with mining activity in a semi-arid region. *J. Environ. Manag*. **273** (2020). 10.1016/j.jenvman.2020.111148 32758915

[3] Kumar, V. *et al*. Assessment of heavy-metal pollution in three different Indian water bodies by combination of multivariate analysis and water pollution indices. *Human Ecol. Risk Assessment Int. J*. **26**, 1–16 (2020).

[4] Statistical Yearbook of Urban and Rural Construction. https://www.ndrc.gov.cn/fggz/hjyzy/sjyybh/202111/t20211105_1303101.html?code=&state=123 (2020). Accessed 20 May 2022.

[5] National Action Plan for Water Conservation. http://www.gov.cn/gongbao/content/2019/content_5419221.htm (2019). Accessed 20 May 2022.

[6] Mao Z, Xue X, Tian H, Michael AU (2019). How will China realize SDG 14 by 2030? A case study of an institutional approach to achieve proper control of coastal water pollution. J. Environ. Manage..

[7] Zhou Z, Liu J, Zhou N, Zhang T, Zeng H (2021). Does the “10-Point Water Plan” reduce the intensity of industrial water pollution? Quasi-experimental evidence from China. J. Environ. Manage..

[8] Zhou Q, Wang Y, Zeng M, Jin Y, Zeng H (2021). Does China's river chief policy improve corporate water disclosure? A quasi-natural experimental. J. Clean. Prod..

[9] Chen, L., Zheng, H. & Yang, H. Rule design and status quo evaluation on cross-industrial wastewater emissions trading in China's typical industrial sectors. *Environ. Sci. Europe*. (2020).

[10] Wang Y, Bian Y, Xu H (2015). Water use efficiency and related pollutants' abatement costs of regional industrial systems in China: A slacks-based measure approach. J. Clean. Prod..

[11] Fujii H, Managi S (2017). Wastewater management efficiency and determinant factors in the Chinese industrial sector from 2004 to 2014. Water.

[12] Yang W, Li L (2017). Efficiency evaluation and policy analysis of industrial wastewater control in China. Energies.

[13] Ren, F.-r., Chen, K.-j., Tian, Z. & Zhang, Y. The investment and treatment efficiencies of industrial solid waste in China’s Yangtze and non-Yangtze River Economic Belts. *J. Mater. Cycles Waste Manag*. **24**, 900–916 (2022).

[14] Sun, Y. *et al*. Evaluation of wastewater pollution and treatment efficiencies in China during urbanization based on dynamic exogenous variable data envelopment analysis. *Front. Environ. Sci*. **11** (2021).

[15] Sun Y-N, Ren F-R, Liu J-W, Shi N-X (2020). Associated effects and efficiency evaluation between wastewater pollution and water disease based on the dynamic two-stage DEA model. Healthcare.

[16] Zhang B, Bi J, Fan Z, Yuan Z, Ge J (2008). Eco-efficiency analysis of industrial system in China: A data envelopment analysis approach. Ecol. Econ..

[17] Dong X, Zhang X, Zeng S (2017). Measuring and explaining eco-efficiencies of wastewater treatment plants in China: An uncertainty analysis perspective. Water Res..

[18] Gémar G, Gómez T, Molinos-Senante M, Caballero R, Sala-Garrido R (2018). Assessing changes in eco-productivity of wastewater treatment plants: The role of costs, pollutant removal efficiency, and greenhouse gas emissions. Environ. Impact Assess. Rev..

[19] Song M, Xie Q, Shen Z (2021). Impact of green credit on high-efficiency utilization of energy in China considering environmental constraints. Energy Policy.

[20] Ding T, Wu H, Jia J, Wei Y, Liang L (2020). Regional assessment of water-energy nexus in China’s industrial sector: An interactive meta-frontier DEA approach. J. Clean. Prod..

[21] Ayyildiz E, Yildiz A, Taskin Gumus A, Ozkan C (2021). An integrated methodology using extended Swara and DEA for the performance analysis of wastewater treatment plants: Turkey case. Environ. Manag..

[22] Wu J, Li M, Zhu Q, Zhou Z, Liang L (2019). Energy and environmental efficiency measurement of China's industrial sectors: A DEA model with non-homogeneous inputs and outputs. Energy Econ..

[23] Sueyoshi, T., Yuan, Y. & Goto, M. A literature study for DEA applied to energy and environment. *Energy Econ*. **62** (2016).

[24] Yang G, Gong G, Gui Q (2022). Exploring the spatial network structure of agricultural water use efficiency in China: A social network perspective. Sustainability.

[25] Wang F, Yu C, Xiong L, Chang Y (2019). How can agricultural water use efficiency be promoted in China? A spatial-temporal analysis. Resour. Conserv. Recycl..

[26] An M (2018). Spatial patterns of urban wastewater discharge and treatment plants efficiency in China. Int. J. Environ. Res. Public Health.

[27] Zhang Q (2016). Current status of urban wastewater treatment plants in China. Environ. Int..

[28] Hernández-Sancho F, Molinos-Senante M, Sala-Garrido R (2011). Techno-economical efficiency and productivity change of wastewater treatment plants: The role of internal and external factors. J. Environ. Monit..

[29] Hernández-Sancho F, Molinos-Senante M, Sala-Garrido R (2011). Energy efficiency in Spanish wastewater treatment plants: A non-radial DEA approach. Sci. Total Environ..

[30] Huang R (2021). Evaluating the energy efficiency of wastewater treatment plants in the Yangtze River Delta: Perspectives on regional discrepancies. Appl. Energy.

[31] Jiang H (2020). Sustainability efficiency assessment of wastewater treatment plants in China: A data envelopment analysis based on cluster benchmarking. J. Clean. Prod..

[32] Zhou X (2018). Assessing integrated water use and wastewater treatment systems in China: A mixed network structure two-stage SBM DEA model. J. Clean. Prod..

[33] Hu Z, Yan S, Yao L, Moudi M (2018). Efficiency evaluation with feedback for regional water use and wastewater treatment. J. Hydrol..

[34] Bian Y, Yan S, Xu H (2014). Efficiency evaluation for regional urban water use and wastewater decontamination systems in China: A DEA approach. Resour. Conserv. Recycl..

[35] Pan, D., Hong, W. & Kong, F. Efficiency evaluation of urban wastewater treatment: Evidence from 113 cities in the Yangtze River Economic Belt of China. *J. Environ. Manag*. 270 (2020). 10.1016/j.jenvman.2020.110940 32721357

[36] Huang Y, Huang X, Xie M, Cheng W, Shu Q (2021). A study on the effects of regional differences on agricultural water resource utilization efficiency using super-efficiency SBM model. Sci. Rep..

[37] Banker RD, Charnes A, Cooper WW (1984). Some models for estimating technical and scale inefficiencies in data envelopment analysis. Manage. Sci..

[38] Charnes A, Cooper WW, Rhodes E (1978). Measuring the efficiency of decision making units. Eur. J. Oper. Res..

[39] Tone K, Tsutsui M (2010). Dynamic DEA: A slacks-based measure approach. Omega.

[40] Shi Z, She Z, Chiu Y-H, Qin S, Zhang L (2021). Assessment and improvement analysis of economic production, water pollution, and sewage treatment efficiency in China. Socioecon. Plann. Sci..

[41] Yang J, Liu X, Ying L, Chen X, Li M (2020). Correlation analysis of environmental treatment, sewage treatment and water supply efficiency in China. Sci. Total Environ..

