# Supplementary material for: A modified dynamic DEA model to assess the wastewater treatment efficiency: perspective from Yangtze River and Non-Yangtze River Basin
Source: Sci Rep. 2022 Jun 15;12:9931. doi: 10.1038/s41598-022-14105-0 (PMC9200827; doi:10.1038/s41598-022-14105-0)
Supplement: Supplementary file 1 — Supplementary Tables. [file 41598_2022_14105_MOESM1_ESM.docx]

**Appendix A**

| **Table 4.** Total efficiency scores and rankings of provinces in Stage 1 | | | | | | | | | | | |  |
| --- | --- | --- | --- | --- | --- | --- | --- | --- | --- | --- | --- | --- |
|  |  | 2013 | | 2014 | | 2015 | | 2016 | | 2017 | |  |
| No. | DMU | Score | Rank | Score | Rank | Score | Rank | Score | Rank | Score | Rank | AVE (five years) |
| 1 | Anhui | 0.3172 | 26 | 0.2361 | 28 | 0.2923 | 26 | 0.2742 | 21 | 0.3035 | 21 | 0.2847 |
| 2 | Zhejiang | 0.4949 | 6 | 0.479 | 7 | 0.5306 | 9 | 0.4709 | 8 | 0.51 | 7 | 0.4971 |
| 3 | Chongqing | 0.38 | 14 | 0.3634 | 11 | 0.725 | 5 | 0.3517 | 14 | 0.3775 | 15 | 0.4395 |
| 4 | Fujian | 0.4096 | 10 | 0.3894 | 9 | 0.4744 | 11 | 0.4565 | 10 | 0.5042 | 8 | 0.4468 |
| 5 | Gansu | 0.3083 | 29 | 0.2144 | 31 | 0.2771 | 28 | 0.222 | 31 | 0.2379 | 30 | 0.2519 |
| 6 | Guangdong | 0.5235 | 4 | 0.5204 | 5 | 0.5635 | 8 | 0.4333 | 11 | 0.4524 | 11 | 0.4986 |
| 7 | Guangxi | 0.3144 | 28 | 0.2597 | 24 | 0.3257 | 24 | 0.4574 | 9 | 0.4624 | 10 | 0.3639 |
| 8 | Guizhou | 0.3059 | 31 | 0.2331 | 29 | 0.282 | 27 | 0.2829 | 20 | 0.2907 | 23 | 0.2789 |
| 9 | Sichuan | 0.3315 | 21 | 0.2797 | 20 | 0.4274 | 14 | 0.2461 | 27 | 0.2677 | 25 | 0.3105 |
| 10 | Shaanxi | 0.3929 | 13 | 0.3005 | 15 | 0.3885 | 18 | 0.3373 | 17 | 0.3681 | 17 | 0.3575 |
| 11 | Shanghai | 1 | 1 | 1 | 1 | 1 | 1 | 1 | 1 | 1 | 1 | 1 |
| 12 | Henan | 0.3341 | 20 | 0.2849 | 18 | 0.4064 | 17 | 0.3499 | 15 | 0.3848 | 14 | 0.352 |
| 13 | Hubei | 0.3506 | 17 | 0.319 | 13 | 0.3838 | 21 | 0.3448 | 16 | 0.3724 | 16 | 0.3541 |
| 14 | Hunan | 0.3304 | 22 | 0.3055 | 14 | 0.3527 | 23 | 0.3112 | 19 | 0.3325 | 19 | 0.3265 |
| 15 | Yunnan | 0.3066 | 30 | 0.2499 | 25 | 0.3883 | 19 | 0.2421 | 28 | 0.2589 | 26 | 0.2892 |
| 16 | Jiangsu | 0.5032 | 5 | 0.5214 | 4 | 0.6298 | 6 | 0.5396 | 5 | 0.6019 | 5 | 0.5592 |
| 17 | Jiangxi | 0.3147 | 27 | 0.2488 | 26 | 0.3096 | 25 | 0.2578 | 24 | 0.294 | 22 | 0.285 |
| 18 | Qinghai | 0.3413 | 18 | 0.2316 | 30 | 0.2699 | 30 | 0.2275 | 30 | 0.2375 | 31 | 0.2616 |
| **YRB AVE score** | | **0.4033** | | **0.3576** | | **0.4459** | | **0.3781** | | **0.4031** | | **0.3976** |
| 19 | Ningxia | 0.3257 | 24 | 0.2966 | 16 | 0.4352 | 12 | 0.2509 | 25 | 0.2797 | 24 | 0.3176 |
| 20 | Hebei | 0.3566 | 16 | 0.287 | 17 | 0.4872 | 10 | 0.3165 | 18 | 0.3504 | 18 | 0.3595 |
| 21 | Jilin | 0.3985 | 12 | 0.321 | 12 | 0.385 | 20 | 0.4317 | 12 | 0.4141 | 13 | 0.3901 |
| 22 | Shandong | 0.4183 | 9 | 0.4069 | 8 | 0.599 | 7 | 0.5066 | 6 | 0.533 | 6 | 0.4928 |
| 23 | Heilongjiang | 0.3353 | 19 | 0.2757 | 22 | 0.4338 | 13 | 0.3653 | 13 | 0.4249 | 12 | 0.367 |
| 24 | Shanxi | 0.3651 | 15 | 0.2447 | 27 | 0.2682 | 31 | 0.2633 | 23 | 0.3169 | 20 | 0.2916 |
| 25 | Inner Mongolia | 0.4728 | 7 | 0.3697 | 10 | 0.4212 | 15 | 0.4717 | 7 | 0.4768 | 9 | 0.4424 |
| 26 | Tianjin | 1 | 1 | 1 | 1 | 1 | 1 | 1 | 1 | 1 | 1 | 1 |
| 27 | Xinjiang | 0.3254 | 25 | 0.2633 | 23 | 0.367 | 22 | 0.2495 | 26 | 0.2543 | 27 | 0.2919 |
| 28 | Beijing | 1 | 1 | 1 | 1 | 1 | 1 | 1 | 1 | 1 | 1 | 1 |
| 29 | Hainan | 0.3281 | 23 | 0.2769 | 21 | 0.4105 | 16 | 0.2374 | 29 | 0.2522 | 28 | 0.301 |
| 30 | Liaoning | 0.4636 | 8 | 0.5041 | 6 | 1 | 1 | 1 | 1 | 1 | 1 | 0.7935 |
| **NYRB AVE score** | | **0.4825** | | **0.4372** | | **0.5673** | | **0.5077** | | **0.5252** | | **0.5040** |

**Appendix B**

**Table 5.** Input costs, wastewater, COD efficiency scores, and rankings for each province and city in Stage 1

| **DMU** | (I) Expense | | | | |  | (OBAD) Wastewater | | | | |  | (OBAD) COD | | | | | |  | |
| --- | --- | --- | --- | --- | --- | --- | --- | --- | --- | --- | --- | --- | --- | --- | --- | --- | --- | --- | --- | --- |
|  | **2013** | **2014** | **2015** | **2016** | **2017** | **AVE** | **2013** | **2014** | **2015** | **2016** | **2017** | **AVE** | **2013** | **2014** | **2015** | **2016** | **2017** | **AVE** | |  |
| Shanghai | 1 | 1 | 1 | 1 | 1 | 1 | 1 | 1 | 1 | 1 | 1 | 1 | 1 | 1 | 1 | 1 | 1 | 1 | |  |
| Jiangsu | 0.0382 | 0.0794 | 0.4079 | 0.1798 | 0.2064 | 0.1823 | 0.624 | 0.6499 | 0.6269 | 0.5564 | 0.5661 | 0.6047 | 0.7775 | 0.7853 | 0.3208 | 0.2575 | 0.2813 | 0.4845 | |  |
| Guangdong | 0.4115 | 0.4099 | 0.4359 | 0.3013 | 0.3141 | 0.3745 | 0.6566 | 0.6304 | 0.7146 | 0.6531 | 0.6816 | 0.6673 | 0.4322 | 0.4301 | 0.5952 | 0.4488 | 0.4184 | 0.4649 | |  |
| Chongqing | 0.0726 | 0.1935 | 0.7385 | 0.3501 | 0.3581 | 0.3426 | 0.5229 | 0.5705 | 0.583 | 0.6905 | 0.6432 | 0.602 | 0.5004 | 0.4968 | 0.3869 | 0.2634 | 0.2417 | 0.3778 | |  |
| Zhejiang | 0.1033 | 0.0483 | 0.225 | 0.1052 | 0.106 | 0.1176 | 0.6119 | 0.6117 | 0.8531 | 0.6093 | 0.6083 | 0.6589 | 0.7172 | 0.7126 | 0.5554 | 0.2978 | 0.2735 | 0.5113 | |  |
| Fujian | 0.0409 | 0.0266 | 0.333 | 0.3178 | 0.3434 | 0.2123 | 0.4921 | 0.5304 | 0.8659 | 0.6822 | 0.7798 | 0.6701 | 0.5249 | 0.5178 | 0.7417 | 0.4244 | 0.3373 | 0.5092 | |  |
| Guangxi | 0.0505 | 0.0324 | 0.3019 | 0.8197 | 0.8395 | 0.4088 | 0.374 | 0.4077 | 0.5495 | 0.6334 | 0.6771 | 0.5283 | 0.2919 | 0.2882 | 0.3698 | 0.3581 | 0.3373 | 0.3291 | |  |
| Shaanxi | 0.046 | 0.027 | 0.4196 | 0.1517 | 0.1577 | 0.1604 | 0.7452 | 0.7167 | 0.6801 | 0.6819 | 0.8 | 0.7248 | 0.4707 | 0.4688 | 0.8023 | 0.5857 | 0.5664 | 0.5788 | |  |
| Henan | 0.0476 | 0.0534 | 0.5643 | 0.2923 | 0.3092 | 0.2534 | 0.4569 | 0.4695 | 0.4266 | 0.4269 | 0.5406 | 0.4641 | 0.364 | 0.3611 | 0.2931 | 0.1887 | 0.1907 | 0.2795 | |  |
| Hubei | 0.0188 | 0.0469 | 0.3057 | 0.1378 | 0.145 | 0.1308 | 0.4916 | 0.5236 | 0.6732 | 0.7822 | 0.6201 | 0.6181 | 0.3593 | 0.3586 | 0.3077 | 0.4831 | 0.4402 | 0.3898 | |  |
| Hunan | 0.0226 | 0.0234 | 0.2288 | 0.0807 | 0.0824 | 0.0876 | 0.4673 | 0.5002 | 0.6376 | 0.6863 | 0.7262 | 0.6035 | 0.3024 | 0.2985 | 0.451 | 0.7382 | 0.6936 | 0.4967 | |  |
| Sichuan | 0.0494 | 0.0189 | 0.1844 | 0.0939 | 0.0946 | 0.0882 | 0.5047 | 0.4895 | 1 | 1 | 1 | 0.7988 | 0.3274 | 0.3197 | 1 | 1 | 1 | 0.7294 | |  |
| Yunnan | 0.0379 | 0.0437 | 0.2484 | 0.2646 | 0.2599 | 0.1709 | 0.4437 | 0.4622 | 0.5207 | 0.4774 | 0.6072 | 0.5022 | 0.3307 | 0.3271 | 0.3849 | 0.3304 | 0.3916 | 0.3529 | |  |
| Anhui | 0.0228 | 0.0214 | 0.2666 | 0.1511 | 0.1626 | 0.1249 | 0.4235 | 0.435 | 0.7932 | 0.6043 | 0.6144 | 0.5741 | 0.326 | 0.3208 | 0.728 | 0.2684 | 0.2624 | 0.3811 | |  |
| Qinghai | 0.0242 | 0.022 | 0.2063 | 0.0684 | 0.0654 | 0.0773 | 0.5877 | 0.5881 | 1 | 1 | 1 | 0.8352 | 0.3095 | 0.2943 | 1 | 1 | 1 | 0.7208 | |  |
| Jiangxi | 0.0261 | 0.0204 | 0.2863 | 0.1061 | 0.1314 | 0.1141 | 0.4056 | 0.4294 | 0.8417 | 0.6911 | 0.6301 | 0.5996 | 0.3009 | 0.2972 | 0.4239 | 0.2392 | 0.2639 | 0.305 | |  |
| Guizhou | 0.0234 | 0.0114 | 0.2058 | 0.2516 | 0.2299 | 0.1444 | 0.5066 | 0.5009 | 0.7083 | 0.5379 | 0.5302 | 0.5568 | 0.378 | 0.3767 | 0.759 | 0.2208 | 0.2375 | 0.3944 | |  |
| Gansu | 0.0358 | 0.0296 | 0.2732 | 0.1451 | 0.1477 | 0.1263 | 0.5742 | 0.5888 | 0.6228 | 0.6082 | 0.6241 | 0.6036 | 0.2551 | 0.2496 | 0.7327 | 0.5687 | 0.6046 | 0.4821 | |  |
| **AVE Score** | **0.1151** | **0.1171** | **0.3684** | **0.2676** | **0.2752** | **0.2287** | **0.5494** | **0.5614** | **0.7276** | **0.6845** | **0.7027** | **0.6451** | **0.4427** | **0.4391** | **0.6029** | **0.4818** | **0.4745** | **0.4882** | |  |
| Beijing | 1 | 1 | 1 | 1 | 1 | 1 | 1 | 1 | 0.4425 | 0.545 | 0.5963 | 0.7168 | 1 | 1 | 0.3187 | 0.2865 | 0.2695 | 0.5749 | |  |
| Tianjin | 1 | 1 | 1 | 1 | 1 | 1 | 1 | 1 | 0.9543 | 0.5542 | 0.5702 | 0.8157 | 1 | 1 | 1 | 0.3829 | 0.37 | 0.7506 | |  |
| Liaoning | 0.0604 | 0.3886 | 1 | 1 | 1 | 0.6898 | 0.8443 | 0.7993 | 0.5884 | 0.6414 | 0.7125 | 0.7172 | 0.4158 | 0.4141 | 0.5271 | 0.4174 | 0.4013 | 0.4351 | |  |
| Shandong | 0.0447 | 0.0591 | 0.8088 | 0.2906 | 0.3037 | 0.301 | 0.6611 | 0.6564 | 0.4356 | 0.5271 | 0.4681 | 0.5497 | 0.4562 | 0.4549 | 0.296 | 0.2764 | 0.202 | 0.3371 | |  |
| Inner Mongolia | 0.0619 | 0.023 | 0.3126 | 0.0817 | 0.0814 | 0.1121 | 1 | 0.9744 | 0.8349 | 0.6846 | 0.6259 | 0.824 | 0.2762 | 0.2751 | 0.2797 | 0.3001 | 0.3136 | 0.2889 | |  |
| Jilin | 0.1369 | 0.0315 | 0.22 | 0.3658 | 0.29 | 0.2088 | 0.6697 | 0.642 | 0.4832 | 0.5127 | 0.5571 | 0.5729 | 0.2591 | 0.2532 | 0.3619 | 0.4974 | 0.5123 | 0.3768 | |  |
| Hebei | 0.0667 | 0.0687 | 0.3756 | 0.3568 | 0.4066 | 0.254 | 0.5675 | 0.5396 | 0.5296 | 0.5567 | 0.575 | 0.553 | 0.3247 | 0.3161 | 0.2981 | 0.3012 | 0.2918 | 0.3064 | |  |
| Ningxia | 0.0389 | 0.3043 | 0.4929 | 0.2539 | 0.2796 | 0.2739 | 0.3988 | 0.4195 | 0.9315 | 0.9117 | 0.9081 | 0.7139 | 0.1763 | 0.1706 | 0.2647 | 0.6355 | 0.645 | 0.3784 | |  |
| Heilongjiang | 0.0389 | 0.0528 | 0.1927 | 0.2864 | 0.266 | 0.167 | 0.5964 | 0.5848 | 1 | 1 | 1 | 0.8362 | 0.1482 | 0.1423 | 1 | 1 | 1 | 0.6581 | |  |
| Xinjiang | 0.0252 | 0.0507 | 0.1889 | 0.2127 | 0.194 | 0.1343 | 0.4888 | 0.5176 | 0.8197 | 0.6043 | 0.6672 | 0.6195 | 0.1926 | 0.1877 | 0.3945 | 0.1462 | 0.165 | 0.2172 | |  |
| Hainan | 0.0281 | 0.0565 | 0.1981 | 0.0652 | 0.0649 | 0.082 | 0.5124 | 0.512 | 0.5906 | 0.5021 | 0.4738 | 0.518 | 0.2507 | 0.242 | 0.2878 | 0.2137 | 0.2279 | 0.2444 | |  |
| Shanxi | 0.0837 | 0.0491 | 0.1514 | 0.1849 | 0.2233 | 0.138 | 0.6476 | 0.5541 | 0.6065 | 0.5949 | 0.6394 | 0.6085 | 0.3863 | 0.3743 | 0.4641 | 0.597 | 0.5518 | 0.4747 | |  |
| **AVE Score** | **0.2155** | **0.257** | **0.4951** | **0.4248** | **0.4258** | **0.3636** | **0.6989** | **0.6833** | **0.6847** | **0.6362** | **0.6495** | **0.6705** | **0.4072** | **0.4025** | **0.4577** | **0.4212** | **0.4125** | **0.4202** | |  |
